# Context and scale: Distinctions for improving debates about physician “rationing”

**DOI:** 10.1186/s13010-017-0048-6

**Published:** 2017-08-29

**Authors:** Jon C. Tilburt, Daniel P. Sulmasy

**Affiliations:** 1 College of Medicine, Mayo Clinic, 200 First Street SW, Rochester, MN 55905 USA; 2 Division of General Internal Medicine, Mayo Clinic, 200 First Street SW, Rochester, MN 55905 USA; 3 Biomedical Ethics Program, Mayo Clinic, 200 First Street SW, Rochester, MN 55905 USA; 4 Health Care Policy and Research, Mayo Clinic, 200 First Street SW, Rochester, MN 55905 USA; 5 Departments of Philosophy and Medicine, Georgetown University, 37th and O Streets NW, Washington, DC, 20057 USA; 6 Pellegrino Center for Clinical Bioethics, Georgetown University, 37th and O Streets NW, Washington, DC, 20057 USA; 7 The Kennedy Institute of Bioethics, Georgetown University, 37th and O Streets NW, Washington, DC, 20057 USA

**Keywords:** Ethics, Decision making, Access to care, Professionalism

## Abstract

Important discussions about limiting care based on professional judgment often devolve into heated debates over the place of physicians in bedside rationing. Politics, loaded rhetoric, and ideological caricature from both sides of the rationing debate obscure precise points of disagreement and consensus, and hinder critical dialogue around the obligations and boundaries of professional practice. We propose a way forward by reframing the rationing conversation, distinguishing between the scale of the decision (macro vs. micro) and its context (ordinary allocation vs. extraordinary re-allocation) avoiding the word “rationing.” We propose to shift the terminology, using specific, descriptive words to defuse conflict and re-focus the debate towards substantive issues. These distinctions can clarify the real ethical differences at stake and facilitate a more constructive conversation about the clinical and social responsibilities of physicians to use resources ethically at the bedside and their role in allocating medical resources at a societal level.

Physicians have never performed all possible tests and treatments for patients and, in this sense, have always limited care based on professional judgment. Yet questions of whether they ought not to perform indicated tests or treatments they deem too costly have vexed the profession for decades [1]. Disagreements about these practices are often framed in terms of the “rightness” or “wrongness” of “bedside rationing.” [2].

**Table 1:**
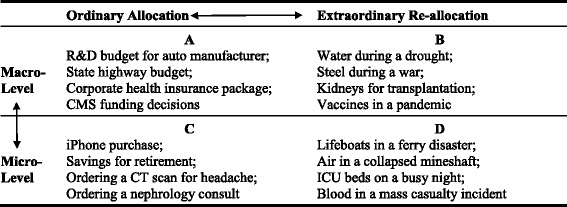
Ordinary Allocation and Extraordinary Re-Allocation on Macro and Micro Decision Making Levels with Examples

In circumstances such as triaging ICU beds in a disaster, all agree bedside rationing may be necessary. Beyond that, reasoning about its ethical legitimacy diverges dramatically. Advocates for a broader role for physicians in rationing argue that costs are unsustainable, that resources are limited, that we already limit care in various ways, and that since physicians can’t avoid limiting care, “rationing at the bedside” is not really optional, but obligatory [2]. These advocates are not arguing for capricious resource distribution or inhumane care. Rather, they argue that, even if it causes some angst and inner conflict, as long as they do so fairly, individual physicians must help ameliorate society’s looming health care resource crisis through individual patient decisions [3]. For bedside rationing advocates, the ethical question is not *whether* to ration, but *how* to ration responsibly.

Opponents of bedside rationing argue that “rationing” at the bedside runs contrary to the traditional values of medicine, and that doing so is not the physician’s job [4]. Rather, a physician’s responsibility to society is secondary to a primary obligation to the individual patient [4]. Opponents are neither ignorant of the healthcare cost crisis nor opposed to setting limits, but they balk at its broader application, because doing so risks violating their primary professional calling [5, 6]. For bedside rationing opponents the ethical question is not *how* bedside rationing should be done responsibly, but whether it is *ever* permissible outside of the rare circumstances cited above.

This debate, unfortunately, has often been marred by politics, loaded rhetoric, and ideological caricature [7], leaving the general public and the profession bewildered, skeptical, and polarized.

We propose a way forward by reframing the rationing conversation that avoids the loaded nature of the word itself. We prefer the word “allocation” and outline distinctions in allocation scale and context. With respect to scale, we note that allocation takes place along a spectrum of scale. When an individual agent makes an allocation decision affecting another individual, the scale remains micro. When a collective agent, like a government makes an allocation decision for a large group, the scale is macro. With respect to context, we draw a distinction between the making of ordinary allocational decisions in the context of relative abundance, and the extraordinary re-allocation of resources that takes place in contexts in which grave shortages are foregrounded. In the latter case, an important resource is acutely limited and re-allocation among potential beneficiaries is deemed necessary. These two distinctions―scale and context―can clarify the real ethical differences at stake and enable a more substantive debate about the clinical and social responsibilities of physicians at the bedside and in the wider society.

## Context: Ordinary allocation vs. extraordinary re-allocation

The verb ‘to allocate’ can be used to describe how resources (which are always, by their nature, limited) are deployed. Allocation takes place in different contexts, however, depending on the relative scarcity of resources, impact on potential beneficiaries, and the intention of the decision maker. At one extreme, *ordinary allocation* decisions occur on a routine, ongoing basis by individuals or groups regarding how to use general resources that are only *relatively* limited―an ongoing fact of daily life. Ordinary allocation implies lower acuity and only relative scarcity. Typically, ordinary allocation occurs in governments, organizational, or individual roles established through legitimate procedures. Those decisions are prioritized democratically or ad hoc*.*

By contrast, *extraordinary re-allocation* represents the other end of the contextual spectrum: when decision making involves an immediate, severe, consequential shortage of a *specific* good with no comparable alternative, usually in exceptional circumstances, with a risk of imminent dire consequences for the common good. In this context, a good which would normally be available to a wide swath of eligible potential beneficiaries is withheld from some for the sake of others. Typically, extraordinary re-allocation requires shared, explicit criteria to guide it. For instance, if shared, legitimate criteria are used to distribute a transplantable organ, it can be withheld justifiably from one and given to another. We accept rules of extraordinary re-allocation when there is a consequential, apparent, and severe shortage of a specific good without comparable alternatives.

## Scale: Macro vs. micro decisions

Allocation is also said to take place on either a macro or a micro level, concepts well-known to economists. The scale of an allocation decision can be thought of as the combination of the individual vs. collective nature of the agent doing the allocation as well as the size of the affected population. On the macro scale, governments, private organizations, and others must make decisions in an ongoing way about how best to allocate general resources such as time and money (macro, ordinary allocation) for large groups. The NIH budget is an example of ordinary allocation on the macro level. These same organizational agents may also face acute decisions about how best to distribute a specific irreplaceable resource in a shortage, such as distributing ciprofloxacin supplies after an anthrax attack (macro, extraordinary re-allocation).

Ordinary allocation and extraordinary re-allocation also occur closer to the individual end of the spectrum, i.e., on the“micro” scale. Individual citizens must make ordinary allocation decisions on the micro scale about how they will spend their time, money, and attention—such as whether to purchase an iPhone versus buy new tires. In routine patient care, individual physicians are constantly making ordinary micro-allocation decisions, such as whether to order a CT for headache or a nephrology consult for proteinuria. Decision makers at a micro level will also sometimes face a different context: a severe, consequential shortage of an important, specific resource with no alternatives. For example, the father of a starving family gives up his food for the benefit of his wife and children, or a ferry captain decides how to use lifeboats in a disaster, or an attending allocates ICU beds on a very busy night. These are circumstances of extraordinary re-allocation on a micro level.

Micro decisions sum up to macro effects, but their locus, motives, and scope are micro in scale. Micro and macro also encompass a spectrum characterizing how agency (collective or individual) and impact (large scale or small scale) are experienced by the decision makers.

## Implications for debates about health care rationing

These distinctions about the context and scale of allocation decisions can be depicted in a simple figure that might help clarify ethical debates about physicians and health care rationing (Table 1). This figure is presented as a categorical 2 × 2 table for illustrative purposes, but we fully acknowledge that this is a spectrum of scale and a spectrum of context. The double arrows illustrate that reality. Using this figure, any given circumstance can be plotted on a spectrum of context from ordinary allocation to extraordinary re-allocation as well as a spectrum of scale from macro to micro. Merely categorizing the context and scale of an allocation decision can be a step forward, offering more precision in ethical debates that typically blur these distinctions.

Classifying specific cases could prompt questions. For example, one could ask if there is a morally significant difference between ordinary allocation and extraordinary re-allocation. Are the priority of ethical norms governing decision-maker responsibilities the same everywhere on the fig. (A, B, C, and D)? Why or Why not? Do individual practitioners only functioning near the micro end of the spectrum (C, D) have different obligations under circumstances of exceptional re-allocation (D) than under conditions of routine allocation (C)? What is the ethically proper role for physicians as moral agents in matters pertaining to more macro organizational or societal levels (A, B)? Does the failure of society to devise a fair system of macro ordinary allocation (A) change the nature of physicians’ ordinary allocation obligations on the micro level (C)? Do both exceptional re-allocation and routine allocation deserve the label, “rationing,” or should the term “rationing” be reserved for exceptional re-allocation? At the extreme ends of the context and scale spectrums, the obligations at stake for individual physician may seem clear. Cases in which the context is ambiguous and the scale of the decision is neither clearly macro nor micro will prompt further discussion and debate.

## Conclusions

Debates about health care rationing fit squarely in our broader conversation about justice, and in a pluralistic society, we must first stipulate “whose justice” we are talking about [6]. Nevertheless, these distinctions could help bring more nuance to the conversation about the ethics of bedside rationing, using less loaded terms. Whether one endorses a narrow definition of rationing or a broader one as more ethically justifiable largely reflects some combination of one’s intuitions about the scope of a physician’s professional obligations to individuals (micro) relative to society (macro). Prioritizing substantive distinctions over rhetoric could move debate beyond the “R word” to stimulate the tough but productive conversations these issues require.

## Abbreviations

CT: Computed tomography
ICU: Intensive care unit
NIH: National Institutes of Health
